# What Will the Chancellor of the Exchequer Do? The Two Alternatives

**Published:** 1911-10-14

**Authors:** 


### The Hospital

The Workers' Newspaper of Administrative Medicine and Institutional Life, Administration, National Insurance and Health.

No. 1318, Vol. LI. SATURDAY, OCTOBER 14TH, 1911. PRICE ONE PENNY.

### WHAT WILL THE CHANCELLOR OF THE EXCHEQUER DO? THE TWO ALTERNATIVES.

The Chancellor of the Exchequer between now and the reassembling of Parliament will stand at the parting of the ways. Is he to go down to history as a great statesman who conceived and carried through a scheme of national insurance, which protected all existing interests and united all agencies for the relief of sickness and the preservation of health, so that the United Kingdom became under it almost ideal in the completeness of the facilities afforded to all classes to minimise the evils of disease and to maintain the maximum of individual and national health? Or is he to be remembered as a man who had a high ideal but did not reach it, because he failed to realise the immensity of the interests touched by his scheme of insurance and omitted to avail himself of the very centre and heart of existing agencies for medical treatment—our present system of voluntary hospitals on which the nation has to depend for medical aid and education, the protection and welfare of the patient, and the development of scientific treatment for the defence of all classes of the community? It is perfectly certain that any scheme of national insurance which does not include provision for hospital benefit must fail in practice. Without the amendment to Clause 15 of Mr. Lloyd George's Bill, and the consequent amendments which we suggested last week, the voluntary hospital system must be permanently injured and ultimately destroyed.

To destroy this system would be a grievous wrong to the poor of all classes who use the voluntary hospitals at the present time, and it must bring failure upon Mr. Lloyd George's scheme of national insurance, because financially the cost of the only alternative, State hospitals, would necessitate an increase in taxation of 8s. 6d. per head of the population. To state these alternatives and their consequences tersely and clearly is to convince all prudent people, and especially those who believe, as we do, in the honesty of purpose and enthusiasm of Mr. Lloyd George, that the Bill must be amended by the inclusion of hospital benefit, whereby the danger of excessive expenditure on buildings for sanatoria will be effectually removed.

To strengthen the voluntary hospital system will make Mr. Lloyd George's name live in history as a great statesman, for in this way he can readily effect an enormous improvement in the actual provision for the sick of all classes throughout the United Kingdom. By the inclusion of hospital benefit in the National Insurance Bill and the adjustment of the differences which seem well on the way to a happy solution between the medical profession and the friendly societies, we consider that the passing of Mr. Lloyd George's scheme will be assured. Then the momentous changes which must follow, if statesmen and the people are wise and prudent, will result in one great organised administration which will remove existing abuses, prevent overlapping, and secure and provide the maximum facility for all classes to obtain the hospital, medical, and other care which each individual may require when overtaken by accident or disease.

The voluntary hospitals are contributing considerable aid to the successful realisation of these desirable ends by organising themselves and the opinion of their supporters throughout the United Kingdom. The committees which are now being set up cannot fail, if Mr. Lloyd George decides to include hospital benefit, as we have confidence he will do, to bring together in every locality all the existing agencies for the relief of sickness, the prevention of disease, and the maintenance of public health. Thus we shall arrive by the spontaneous and zealous action of the men who know most about these subjects, and who are at present responsible for the burden of the work which they entail, at an initial organisation upon which the Government can build at the minimum cost a perfected system providing for the medical and health requirements of all. We say the minimum cost, because co-operation and inter-communication between existing authorities and the formation locally of organised committees must secure the prevention of overlapping, the relegation of each patient to the particular institution or agency which is charged with the duty of providing for him when ill, and the setting free of a number of existing buildings, while it will save the cost of duplicated staffs and a great waste of energy arising at present from a want of combination and that fulness of knowledge which united action and inter-communication must supply.

In making these statements, it must be remembered that they rest upon an experience of forty years' hard work, covering practically every field of the services to which they relate, and that they are based upon an intimate personal knowledge of the actual condition of affairs as they exist to-day, of the evils and the causes of the evils which at present prevail, together with the practical possibility of securing the maximum results foreshadowed in this article. We appreciate the courage, patience, and wholehearted desire which we believe animate Mr. Lloyd George; we desire to help him to secure the most practical success for his insurance scheme; and we make bold to say that, if the present opportunity in regard to the voluntary hospitals is appreciated and taken advantage of by the Chancellor of the Exchequer, within the next decade our forecast of the fruits of his wisdom and statesmanship will be realised.

